# Outcomes in Clinical Subgroups of Patients With Alcohol-Related Hospitalizations

**DOI:** 10.1001/jamanetworkopen.2023.53971

**Published:** 2024-01-31

**Authors:** Erik L. Friesen, Andrea Mataruga, Nathan Nickel, Paul Kurdyak, James M. Bolton

**Affiliations:** 1 Temerty Faculty of Medicine, University of Toronto, Toronto, Ontario, Canada; 2 Centre for Addiction and Mental Health, Toronto, Ontario, Canada; 3 Mental Health and Addictions Research Program, ICES Central, Toronto, Ontario, Canada; 4 Manitoba Centre for Health Policy, Winnipeg, Manitoba, Canada; 5 Department of Community Health Sciences, Max Rady College of Medicine, University of Manitoba, Winnipeg, Manitoba, Canada; 6 Department of Psychiatry, Temerty Faculty of Medicine, University of Toronto, Toronto, Ontario, Canada; 7 Department of Psychiatry, Max Rady College of Medicine, University of Manitoba, Winnipeg, Manitoba, Canada

## Abstract

**Importance:**

Alcohol-related hospitalizations are common and associated with significant cost to the health care system. We have a limited understanding of the characteristics of individuals who experience alcohol-related hospitalizations, which limits our capacity to prioritize those at the highest risk of postdischarge harm.

**Objective:**

To identify and characterize the clinical subgroups of individuals who are hospitalized for alcohol-related harms.

**Design, Setting, and Participants:**

This cohort study used latent class analysis (LCA) to identify clinical subgroups of individuals experiencing alcohol-related hospitalizations in 2 provinces in Canada. All individuals between ages 10 and 105 years who were hospitalized for an alcohol-related harm between January 2017 and December 2018 (ie, the index hospitalization) were eligible. Data were analyzed between June 2023 and August 2023.

**Exposures:**

The exposure of interest was the clinical subgroup that an individual belonged to. These subgroups were identified using an LCA based on (1) the characteristics of the index hospitalization and (2) the history of alcohol-related health service use.

**Main Outcomes and Measures:**

In-hospital mortality, alcohol-related hospital readmission, and all-cause mortality in the year following discharge from the index hospitalization. The association between subgroup membership and the risk of in-hospital and postdischarge outcomes was evaluated using multivariable regression.

**Results:**

A total of 34 043 individuals were included in analysis, 4753 from Manitoba (median [IQR] age, 49 [40-58] years; 1786 female [37.6%]) and 29 290 from Ontario (median [IQR] age, 57 [45-67] years; 8527 female [29.1%]). Seven subgroups were identified following a gradient from low-frequency service use for acute intoxication to high-frequency service use for severe alcohol use disorder and liver disease. In Ontario, there were 4431 individuals in the liver disease subgroup representing 15.5% of the cohort who were at the highest risk of 1-year mortality (1382 [31.2%]) relative to the acute intoxication subgroup (42 [4.0%]) (adjusted hazard ratio [aHR], 3.83; 95% CI, 2.80-5.24). There was also a small subgroup (10.6%) of individuals with high-frequency alcohol-related health service use who had a much higher hazard of readmission following the index hospitalization (1-year readmission: 703 of 1526 [46.1%] vs 104 of 1058 [9.8%] in the acute intoxication subgroup; aHR, 5.09; 95% CI, 4.11-6.31).

**Conclusions and Relevance:**

In this population-based cohort study of individuals experiencing alcohol-related hospitalizations, we identified several small, clinically distinct subgroups that were at a disproportionately high risk of readmission and mortality. These groups could merit prioritization in strategies aimed at reducing the risk of adverse outcomes following alcohol-related hospitalizations.

## Key Points

**Question:**

Are there clinically distinct subgroups of individuals who are hospitalized for alcohol-related harms?

**Findings:**

In this cohort study, we identified 7 unique subgroups of individuals who were hospitalized for alcohol-related harms. These subgroups had different risks of adverse outcomes, with several subgroups, particularly individuals with liver disease and high frequency alcohol-related health service use, being at the highest risk of short-term readmission and mortality.

**Meaning:**

These results suggest that individuals who experience alcohol-related hospitalizations are not a homogeneous population, and a small subset of this cohort may require prioritization in efforts to reduce high rates of short-term readmission and death.

## Teaser

This cohort study of records of alcohol-related hospitalizations from 2 Canadian provinces examines associated risks of adverse outcomes by clinical characteristics and history of alcohol-related health service use.

## Introduction

Alcohol use is a leading cause of death and disability worldwide, precipitating short-term harms associated with acute intoxication and long-term harms associated with chronic alcohol use disorder (AUD).^1^ Alcohol-related hospitalizations are common among individuals with AUD and incur a large cost to health care systems around the world.^1,2,3^ As a result, there have been increasing efforts to understand the epidemiology of alcohol-related hospitalizations using population-based health administrative data.^4,5,6,7,8,9^ Identifying alcohol-related hospitalizations in these large data sets requires the use of indicators for alcohol-related harms, which are sets of diagnostic codes used to identify hospital records that are attributable to alcohol use.^4,9,10^ Popular indicators include those developed by the Canadian Institute for Health Information (CIHI)^11^ and the Centers for Disease Control and Prevention (CDC).^12^

Importantly, these indicators contain many distinct diagnostic categories (eg, the CIHI indicator contains 52 *International Classification of Disease, Tenth Edition* [*ICD-10*] and 10 *Diagnostic and Statistical Manual, Fifth Edition* [*DSM-V*] diagnostic codes^11^), which capture a variety of different clinical populations. Furthermore, some of the individuals that are captured (eg, someone with a first-time hospitalization for alcohol poisoning) may not meet the *DSM-V* criteria for an AUD.^13^ In turn, using this type of composite indicator to evaluate trends in alcohol-related health service use in a population with supposed AUD may fail to consider distinct clinical subgroups that have unique patterns of alcohol-related service use and differential risk of adverse outcomes. This is problematic because it limits our understanding of the individuals who present at a hospital for alcohol-related health conditions and our capacity to identify and assist those who are at the highest risk of downstream harm.

The purpose of this study was to address this gap in knowledge through 3 objectives. The first was to determine if there are distinct clinical subgroups among individuals who are hospitalized for alcohol-related harms based on (1) their presenting diagnosis and (2) their history of alcohol-related health service use. The second was to understand the differences between these subgroups, in terms of sociodemographic correlates and risk of adverse in-hospital and postdischarge outcomes. The third was to replicate the study in 2 independent populations to examine whether these clinical subgroups and outcomes were consistent across jurisdictions.

## Methods

### Study Design

This was a population-based retrospective study of individuals who had an alcohol-related hospitalization between January 1, 2017, and December 31, 2018 (ie, the index hospitalization), in either Ontario or Manitoba, Canada. These individuals were identified using population-based health administrative databases, which capture virtually all hospitalizations in each province. The databases used to capture all study variables are outlined in eTable 1 in Supplement 1. Data sets were linked using unique encoded identifiers and analyzed at ICES (Ontario) and the Manitoba Centre for Health Policy (MCHP). The diagnostic codes used to identify alcohol-related hospitalizations in these databases are those outlined in the CIHI indicator “Hospitalizations Entirely Caused by Alcohol.”^3^ There was a 1-year follow up from the date of discharge from the index hospitalization to collect data on postdischarge outcomes and a 2-year lookback for previous alcohol-related health service use (eFigure 1 in Supplement 1).

For individuals with more than 1 alcohol-related hospitalization during the accrual window, 1 was chosen at random to be the index event, as described previously.^14,15^ Briefly, this serves to prevent against a clustering of index events at the beginning of the accrual window and has become standard practice in retrospective analyses of alcohol-related hospitalizations in Ontario.^14^ Exclusion criteria included being a non-Manitoba or non-Ontario resident, having an invalid unique identifier (required for database linkage), being aged over 105 years or under 10 years,^3^ and not being eligible for universal health insurance for the entirety of the study period.

The cohorts created in Manitoba and Ontario were not linked together but rather analyzed in parallel to facilitate comparisons between provinces. The was done purposefully to evaluate the external validity of the subgroups identified by the latent class analysis (LCA), given that Manitoba and Ontario independently govern their health care systems and health data collection. For context, Ontario and Manitoba are 2 adjacent Canadian provinces. While this adjacency results in similarities between provinces, there are also notable demographic differences. For example, Ontario has a much larger population (approximately 15 million) than Manitoba (1.5 million) and fewer people who live in rural areas (13.3% vs 25.3% in Manitoba).^16^ Additional differences in sociodemographic composition between the provinces can be explored using interactive data available through Statistics Canada.^17^ Manitoba and Ontario also independently run their health care systems and alcohol regulation policies, which translates into differences in the relative accessibility of health services and alcohol between provinces.^18,19,20^

ICES is a prescribed entity under Ontario’s Personal Health Information Protection Act (PHIPA). The use of Ontario data in this project is authorized under section 45 of PHIPA and approved by ICES’ Privacy and Legal Office. Section 45 of PHIPA authorizes ICES to collect personal health information, without consent, for the purpose of analysis or compiling statistical information with respect to the management of, evaluation or monitoring of, the allocation of resources to, or planning for all or part of the health system. The Manitoba data came from databases maintained by Manitoba Health and Shared Health and housed at MCHP. Ethics approval for use of this data was obtained from the health research ethics board at the University of Manitoba. This study adheres to the Strengthening the Reporting of Observational Studies in Epidemiology (STROBE) reporting guideline for cohort studies (eAppendix in Supplement 1).

### Exposures

There were 4 exposures of interest in this study: (1) the alcohol-related diagnostic code(s) associated with the index hospitalization (more than 1 code can be recorded in each record), the count of (2) alcohol-related outpatient visits and (3) alcohol-related emergency department (ED) visits, and (4) alcohol-related hospitalizations in the 2 years prior to the admission date of the index hospitalization. These exposures were chosen because the type of harm and frequency of service use are both indicators of the nature and severity of the underlying AUD that contributed to the index hospitalization.^5,15,21^ These variables were input into an LCA to generate subgroups of individuals hospitalized for alcohol-related harms. These subgroups then became the exposure of interest for the multivariable regression models described below.

### Outcomes

There were 3 outcomes of interest in this study: (1) in-hospital mortality, (2) time to first alcohol-related hospital readmission in the year following discharge from the index hospitalization, and (3) time to death in the year following discharge from the index hospitalization.

### Covariates

Covariates that could foreseeably confound the association between exposures and outcomes were chosen a priori based on previous work on alcohol-related health service use in Canada.^4,5,8,14,22,23^ These variables included age, sex, income quintile (measured using after-tax income), rurality (measured using Statistical Area Classification^24^), medical comorbidity (measured using the Johns Hopkins Adjusted Clinical Group system version 10 aggregated diagnosis groups [ADG] score), and psychiatric comorbidity (measured using a previously described psychiatric severity gradient based on psychiatric service use [outpatient, emergency, and inpatient] during the 2-year lookback^25^). Data on race and ethnicity were not available within the data sources used for this study.

### Statistical Analysis

First, an LCA was run using the alcohol-related diagnostic code(s) associated with the index hospitalization and the count of alcohol-related service encounters in the 2 years prior to the index hospitalization, as described above. Models with increasing numbers of classes were built and the bayesian information criterion (BIC) and Akaike information criterion (AIC) were tabulated for each model specification. The final LCA specification was chosen based on the point at which the BIC no longer improved when an additional class was added to the model and the interpretability of classes, as has been done previously.^26^ Classification diagnostics, specifically the average latent class posterior probability, were tabulated for the final model specification.

For the purposes of describing the clinical and demographic features of the subgroups, individuals were assigned to subgroups based on the class to which they had the highest calculated probability of membership. Descriptive statistics of the primary diagnoses and count of previous alcohol-related health service encounters for each subgroup were tabulated. Sociodemographic and clinical covariates were tabulated after stratifying the cohort by subgroup. Significant differences between subgroups were assessed using χ^2^ tests for independence (categorical variables) and 1-way Kruskal-Wallis analysis of variance (continuous variables).

Finally, the association between subgroup membership and in-hospital mortality was modeled using a multivariable logistic regression model. The association between subgroup membership and time to alcohol-related hospital readmission was modeled using a multivariable Fine-Grey subdistribution hazard model with all-cause mortality included as a competing risk. The association between subgroup membership and time to postdischarge mortality was modeled using a multivariable Cox proportional hazard model. The proportional hazards assumption was evaluated by visualizing for violations in the Kaplan-Meier curves. When modeling postdischarge outcomes, individuals who died prior to discharge from the index event were excluded. Given that subgroup membership is inherently uncertain, subgroup membership was not assigned based on the highest calculated probability of membership in the statistical models. Instead, we used a proportional assignment approach that allows each individual to contribute to each subgroup, weighted based on the posterior probability of belonging to the subgroup. This approach has been validated in recent studies and performs well for modeling the association between latent classes a distal time-to-event outcomes.^27,28^ Each model was adjusted for all covariates listed above. For completeness, unadjusted models were also run.

The LCA was run using the poLCA package in R version 4.2.2 (R Project for Statistical Computing). All other analyses were run in SAS version 9.4 (SAS Institute Inc). The threshold of significance for this study was *P* < .05 and all statistical analyses were 2-tailed.

## Results

### Subgroups Identified by the LCA

**Figure 1.**
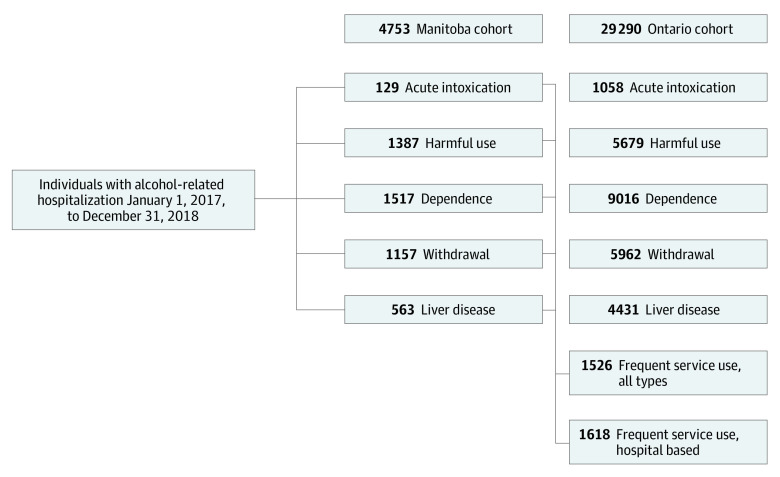
Results From the Latent Class Analysis (LCA) In a cohort of individuals who experienced alcohol-related hospitalizations between January 1, 2017, and December 31, 2018 (the index hospitalization), 5 distinct subgroups of individuals were identified in Manitoba and 7 distinct subgroups were identified in Ontario. The two additional frequent service use subgroups identified in Ontario had a variety of presenting diagnoses (ie, were drawn from the other five subgroups) and were characterized by having the highest frequency of alcohol-related health service use in the 2 years prior to the index hospitalization (see Table 1).

A total of 4753 individuals were included in the Manitoba cohort (median [IQR] age, 49 [40-58] years; 1786 female [37.6%]) and 29 290 individuals were included in the Ontario cohort (median [IQR] age, 57 [45-67] years; 8527 female [29.1%]). A tabulation of individuals excluded from the cohorts by reason is presented in eTable 2 in Supplement 1. A summary of the LCA findings from both provinces is presented in Figure 1. In Manitoba, the LCA found that a model with 5 classes was the best fit (eTables 3 and 4 in Supplement 1). The characteristics of these 5 subgroups in terms of the index code(s) and frequency of previous alcohol-related health service use is presented in eTable 6 in Supplement 1. Based on these characteristics, the subgroups were categorized as follows: (1) individuals presenting with acute intoxication and a low average frequency of prior alcohol-related health service use (129 individuals [2.7%]); (2) individuals presenting with harmful alcohol use, relatively few alcohol-related comorbidities (eg, pancreatitis, liver disease, psychosis), and a low frequency of prior alcohol-related health service use (1387 [29.2%]); (3) individuals with alcohol dependence, more alcohol-related comorbidities, and an average frequency of prior alcohol-related health service use (1517 [31.9%]); (4) individuals presenting for withdrawal with a high average frequency of prior alcohol-related health service use (1157 [24.3%]); and (5) individuals presenting for alcohol-related liver disease with the highest frequency of prior alcohol-related health service use (563 [11.8%]) (Figure 1).

In Ontario, the LCA found that a model with 7 classes was the best fit (eTables 3 and 5 in Supplement 1). The characteristics of these 7 subgroups in terms of the index code(s) and frequency of previous alcohol-related health service use are outlined in Table 1. The first 5 subgroups mirrored those identified in Manitoba, with 1058 individuals (3.6% of the cohort) in the acute intoxication subgroup, 5679 (19.4%) in the harmful use subgroup, 9016 (30.8%) in the alcohol dependence subgroup, 5962 (20.4%) in the withdrawal subgroup, and 4431 (15.1%) in the liver disease subgroup (Figure 1). Two additional groups emerged that were characterized by a variety of presenting health conditions (ie, drawn from the first 5 categories) and the highest frequency of prior alcohol-related health service use. One of which had a high frequency of all types of alcohol-related health service use (ie, outpatient, ED, and inpatient, representing 5.2% of cohort) and the other had a high frequency of prior alcohol-related ED visits and hospitalizations but a less frequent prior alcohol-related outpatient visits (hospital-based, 5.5% of cohort).

**Table 1.** Index Diagnoses and Prior Alcohol-Related Health Service Use Across the 7 Subgroups Identified in Ontario

Characteristic	Individuals with prior alcohol-related service use, No. (%)^a^						
	Acute intoxication (n = 1058)	Harmful use (n = 5679)	Alcohol dependence (n = 9016)	Withdrawal (n = 5962)	Liver disease (n = 4431)	Frequent use—all types (n = 1526)	Frequent use—hospital-based (n = 1618)
Outpatient visits							
0	923 (87.2)	4972 (87.6)	7864 (87.2)	4713 (79.1)	3699 (83.5)	0	828 (51.2)
1	53 (5.0)	406 (7.2)	663 (7.4)	697 (11.7)	401 (9.1)	0	457 (28.2)
≥2	82 (7.8)	301 (5.3)	489 (5.4)	552 (9.3)	331 (7.5)	1526 (100)	333 (20.6)
Emergency department visits							
0	795 (75.1)	4674 (82.3)	7374 (81.8)	4267 (71.6)	3732 (84.2)	0	553 (34.2)
1	139 (13.1)	640 (11.3)	1062 (11.8)	979 (16.4)	527 (11.9)	0	323 (20.0)
≥2	124 (11.7)	365 (6.4)	580 (6.4)	716 (12.0)	172 (3.9)	1526 (100)	742 (45.9)
Hospitalizations							
0	924 (87.3)	5006 (88.2)	7896 (87.6)	5049 (84.7)	3179 (71.7)	468 (30.7)	0
1	96 (9.1)	595 (10.5)	1120 (12.4)	913 (15.3)	845 (19.1)	353 (23.1)	0
≥2	38 (3.6)	78 (1.4)	0	0	407 (9.2)	705 (46.2)	1618 (100)
***ICD-10* code**							
F10.0 (Alcohol intoxication)	32 (3.0)	103 (1.8)	1760 (19.5)	125 (2.1)	31 (0.7)	158 (10.4)	115 (7.1)
F10.1 (Harmful alcohol use)	112 (10.6)	5679 (100)	23 (0.3)	577 (9.7)	387 (8.7)	297 (19.5)	309 (19.1)
F10.2 (Alcohol dependence	73 (6.9)	49 (0.86)	3098 (34.36)	551 (9.2)	366 (8.3)	285 (18.7)	331 (20.5)
F10.3 (Alcohol withdrawal)	48 (4.54)	0	NR	5962 (100)	310 (7.0)	829 (54.3)	616 (38.1)
F10.4 (Alcohol withdrawal with delirium)	20 (1.89)	120 (2.11)	1268 (14.06)	53 (0.9)	90 (2.0)	104 (6.8)	94 (5.8)
F10.5 (Alcohol-related psychotic disorder)	NR	NR	45 (0.5)	9 (0.2)	NR	NR	6 (0.4)
F10.6 (Alcohol-related amnesic syndrome)	0 (0)	17 (0.3)	195 (2.16)	35 (0.6)	13 (0.3)	9 (0.6)	40 (2.5)
F10.7 (Alcohol-related residual psychotic disorder)	NR	8 (0.14)	174 (1.93)	21 (0.4)	11 (0.3)	NR	24 (1.5)
F10.8 (Alcohol-related other mental and behavioral disorder)	NR	NR	38 (0.42)	13 (0.2)	NR	10 (0.7)	6 (0.4)
F10.9 (Alcohol-related unspecified mental and behavioral disorder)	10 (0.95)	NR	246 (2.73)	14 (0.2)	12 (0.3)	18 (1.2)	17 (1.1)
G31.2 (Degeneration of nervous system due to alcohol)	NR	19 (0.33)	124 (1.38)	50 (0.8)	177 (4.0)	8 (0.5)	39 (2.4)
G62.1 (Alcoholic polyneuropathy)	NR	11 (0.19)	44 (0.49)	10 (0.2)	6 (0.1)	0	11 (0.7)
G72.1 (Alcoholic myopathy)	NR	NR	12 (0.13)	NR	NR	NR	NR
I42.6 (Alcoholic cardiomyopathy)	0	41 (0.72)	213 (2.36)	40 (0.7)	16 (0.4)	NR	12 (0.7)
K29.2 (Alcoholic gastritis)	0	38 (0.67)	133 (1.48)	47 (0.8)	13 (0.3)	27 (1.8)	26 (1.6)
K70.0 (Alcoholic liver disease)	NR	20 (0.35)	75 (0.83)	27 (0.5)	14 (0.3)	6 (0.4)	NR
K70.1 (Alcoholic fatty liver)	9 (0.85)	112 (1.97)	516 (5.72)	293 (4.9)	252 (5.7)	157 (10.3)	109 (6.7)
K70.2 (Alcoholic hepatitis)	0	NR	7 (0.08)	NR	0	0	NR
K70.3 (Alcoholic liver cirrhosis)	NR	0	0	41 (0.7)	4431 (100)	141 (9.2)	210 (13.0)
K70.4 (Alcoholic hepatic failure)	NR	23 (0.41)	299 (3.32)	57 (1.0)	461 (10.4)	17 (1.1)	121 (7.5)
K70.9 (Alcoholic liver disease unspecified)	NR	27 (0.48)	194 (2.15)	45 (0.8)	26 (0.59)	13 (0.9)	20 (1.2)
K85.2 (Alcohol-induced acute pancreatitis)	NR	224 (3.94)	1265 (14.03)	240 (4.03)	27 (0.61)	99 (6.5)	212 (13.1)
K86.0 (Alcohol-induced chronic pancreatitis)	0	30 (0.53)	202 (2.24)	28 (0.47)	14 (0.32)	13 (0.9)	83 (5.1)
O35.401 (Maternal care for suspected damage to fetus from alcohol use)	0	0	NR	0	0	0	0
O99.301 (Alcohol use complicating pregnancy or childbirth)	0	59 (1.04)	28 (0.31)	NR	0	0	NR
Q86.0 (Fetal alcohol spectrum disorders)	NR	NR	184 (2.04)	0	0	NR	NR
R78.0 (Finding of alcohol in blood)	0	0	9 (0.1)	0	0	0	0
T51.0 (Toxic effect of ethanol)	775 (73.25)	0	0	0	0	6 (0.4)	s
T51.8 (Toxic effect of alcohol–other)	144 (13.61)	0	0	0	0	0	0
T51.9 (Toxic effect of alcohol–unspecified)	365 (34.5)	0	0	0	NR	10 (0.7)	0
X45 (Accidental alcohol poisoning)	571 (53.97)	0	0	0	0	NR	0
X65 (Intentional alcohol poisoning)	96 (9.07)	0	NR	NR	0	NR	0
Y15 (Alcohol poisoning undetermined intent)	32 (3.02)	103 (1.81)	1760 (19.52)	125 (2.1)	31 (0.7)	158 (10.4)	115 (7.1)

Abbreviation: *ICD-10*, *International Statistical Classification of Diseases and Related Health Problems, Tenth Revision*; NR, not reported (<6 individuals).

^a^ *ICD-10* code results do not sum to 100% because individuals can have multiple diagnoses associated with the index hospitalization.

### Demographic and Clinical Characteristics of Subgroups

Descriptive statistics of the study variables, stratified by subgroup, are presented in Table 2 (Ontario) and eTable 7 in Supplement 1 (Manitoba). In Ontario, the median (IQR) age of the cohort was 57 (45-67) years, 8527 (29.1%) were female, 9329 (32.1%) were in the lowest income quintile, 21 239 (73.1%) lived in large metropolitan centers, and 53.4% had a history of health service use for a non-AUD psychiatric diagnosis (Table 2).

**Table 2.** Demographic and Clinical Characteristics of the Ontario Cohort, Stratified by Subgroup

Characteristic	Individuals with prior alcohol-related service use, No. (%)								*P* value^a^
	Acute intoxication (n = 1058)	Harmful use (n = 5679)	Alcohol dependence (n = 9016)	Withdrawal (n = 5962)	Liver disease (n = 4431)	Frequent use (all types) (n = 1526)	Frequent use (hospital-based) (n = 1618)	Overall (N = 29 290)	
Age, median (IQR), y	39 (26-53)	58 (44-69)	61 (43-68)	57 (46-67)	62 (55-69)	49 (38-57)	54 (44-63)	57 (45-67)	<.001
Sex									
Female	536 (50.7)	1733 (30.5)	2636 (29.2)	1465 (24.6)	1226 (27.7)	448 (29.4)	483 (29.9)	8527 (29.1)	<.001
Male	522 (49.3)	3946 (69.5)	6380 (70.8)	4497 (75.4)	3205 (72.3)	1078 (70.6)	1135 (70.1)	20 763 (70.9)	
Income quintile									
1 (lowest)	327 (31.2)	1898 (33.7)	2756 (30.8)	1859 (31.4)	1250 (28.4)	573 (38.0)	666 (41.5)	9329 (32.1)	<.001
2	204 (19.5)	1214 (21.6)	1970 (22.0)	1279 (21.6)	995 (22.6)	323 (21.4)	356 (22.2)	6341 (21.8)	
3	174 (16.6)	978 (17.4)	1630 (18.2)	1072 (18.1)	835 (19.0)	226 (15.0)	237 (14.8)	5152 (17.7)	
4	177 (16.9)	787 (14.0)	1364 (15.2)	893 (15.1)	670 (15.2)	203 (13.4)	189 (11.8)	4283 (14.7)	
5 (highest)	167 (15.9)	752 (13.4)	1229 (13.7)	815 (13.8)	646 (14.7)	185 (12.3)	158 (9.8)	3952 (13.6)	
Statistical area classification									
Large metropolitan	747 (71.14)	4195 (74.49)	6086 (67.97)	4434 (74.89)	3362 (76.43)	1219 (80.73)	1196 (74.47)	21 239 (73.1)	<.001
Medium metropolitan	41 (3.9)	121 (2.2)	364 (4.1)	150 (2.5)	111 (2.5)	33 (2.2)	45 (2.8)	865 (3.0)	
Small metropolitan	132 (12.6)	466 (8.3)	848 (9.5)	467 (7.9)	387 (8.8)	100 (6.6)	138 (8.6)	2538 (8.7)	
Rural (strong MIZ)	69 (6.6)	297 (5.3)	580 (6.5)	351 (5.9)	241 (5.5)	48 (3.2)	55 (3.4)	1641 (5.6)	
Rural (moderate MIZ)	41 (3.9)	258 (4.6)	537 (6.0)	266 (4.5)	200 (4.6)	34 (2.3)	61 (3.8)	1397 (4.8)	
Rural (weak MIZ)	10-20	171 (3.0)	374 (4.2)	167 (2.8)	76 (1.7)	39 (2.6)	74 (4.6)	918 (3.2)	
Remote	<6	124 (2.2)	165 (1.8)	86 (1.5)	20-30	37 (2.5)	37 (2.3)	474 (1.6)	
ADG score, median (IQR)	19 (5-26)	22 (12-33)	23 (12-33)	21 (11-31)	31 (22-41)	32 (21-42)	38 (27-48)	24 (14-36)	<.001
Psychiatric comorbidity									
None	225 (21.3)	2817 (49.6)	4473 (49.6)	2924 (49.0)	2657 (60.0)	155 (10.2)	388 (24.0)	13 639 (46.6)	<.001
Outpatient visit	257 (24.3)	662 (11.7)	1048 (11.6)	496 (8.3)	376 (8.5)	582 (38.1)	689 (42.6)	4110 (14.0)	
Emergency department visit	261 (24.7)	854 (15.0)	1268 (14.1)	851 (14.3)	247 (5.6)	428 (28.1)	243 (15.0)	4152 (14.2)	
Hospitalization	315 (29.8)	1346 (23.7)	2227 (24.7)	1691 (28.4)	1151 (26.0)	361 (23.7)	298 (18.4)	7389 (25.2)	

Abbreviations: ADG, aggregated diagnostic group; MIZ, metropolitan influenced zone.

^a^ Significant differences gauged using χ^2^ tests for independence (categorical) and 1-way Kruskal-Wallis analysis of variance (continuous).

Relative to these overall trends, individuals in the acute intoxication subgroup were proportionately younger (median [IQR] age, 39 [26-53] years), more female (536 [50.7%]), had fewer medical comorbidities (median [IQR] ADG score, 19 [5-26]), and more psychiatric comorbidities (78.7% had prior psychiatric care). Individuals in the harmful use, alcohol dependence, and withdrawal subgroups were similar in terms of demographic and clinical characteristics and aligned with the overall trends. Individuals in the liver disease category were proportionately older (median [IQR] age, 62 [55-69] years), higher income (1250 [28.4%] in the lowest income quintile), had more medical comorbidities (median [IQR] ADG score, 31 [22-41]), and fewer psychiatric comorbidities (40.0% had prior psychiatric care). Individuals in the frequent service use categories were proportionately younger (all types median [IQR] age, 49 [38-57] years; hospital-based median [IQR] age, 54 [44-63] years) and had more medical comorbidities (all types median [IQR] ADG score, 32 [21-42]; hospital-based median [IQR] ADG score, 38 [27-48]). Similar results were observed in Manitoba (eTable 7 in Supplement 1), with the caveat being that the high frequency use subgroups were not observed.

### Association Between Subgroup Membership and Study Outcomes

**Figure 2.**
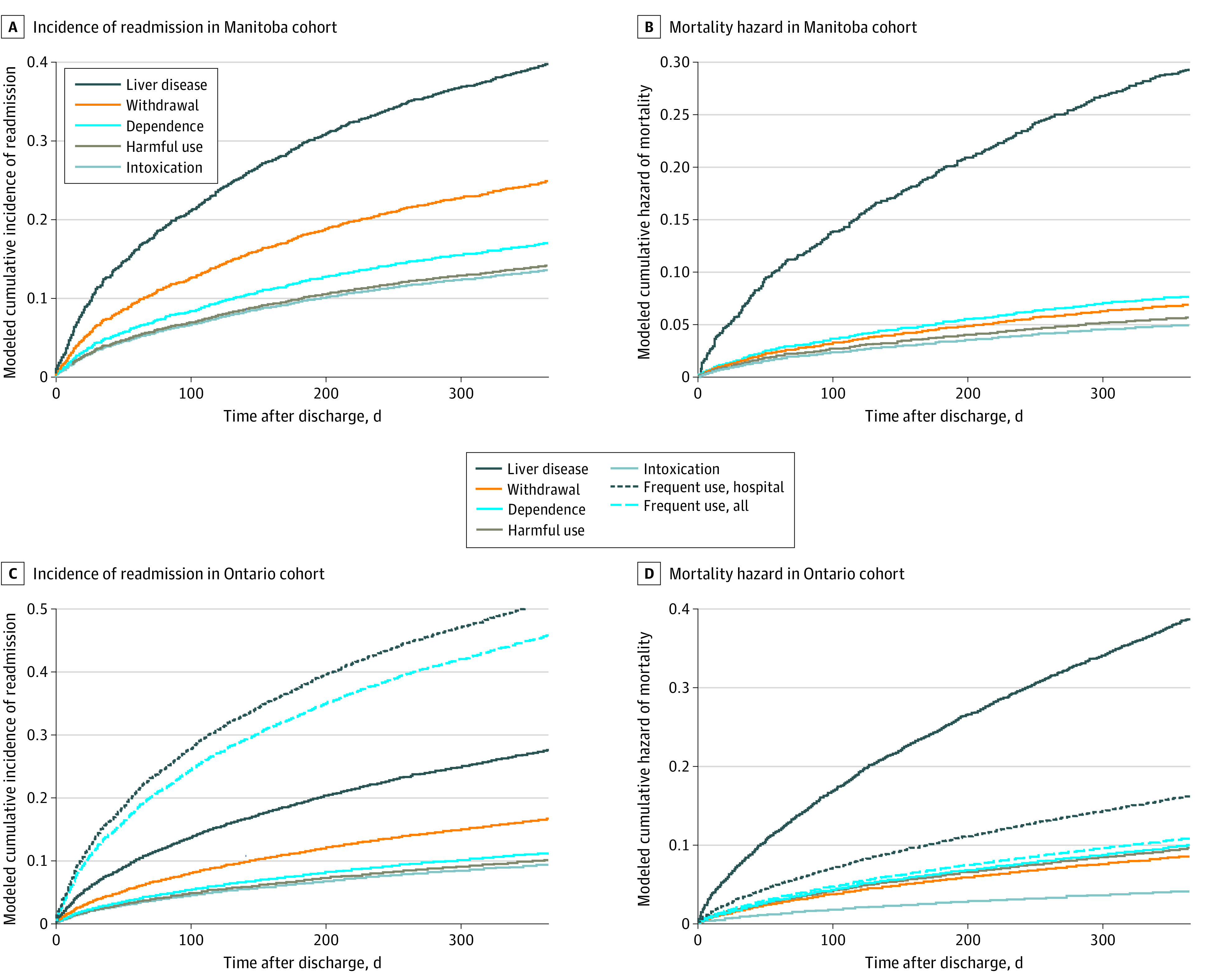
Cumulative Incidence of Readmission and Mortality, Stratified by Subgroup (A) Cumulative incidence (readmission) and hazard (all-cause mortality) curves for outcomes in the year following discharge from the index hospitalization, stratified by subgroup – Manitoba cohort. (B) 1-year incidence of each study outcome, stratified by subgroup – Manitoba cohort. (C) Cumulative incidence (readmission) and hazard (all-cause mortality) curves for outcomes in the year following discharge from the index hospitalization, stratified by subgroup – Ontario cohort. (D) 1-year incidence of each study outcome, stratified by subgroup – Ontario cohort.

The cumulative incidence of alcohol-related hospital readmission and all-cause mortality in the year following discharge from the index hospitalization is presented in Figure 2. The 1-year incidence of these outcomes is available in the supplement (eTables 8 and 9 in Supplement 1). Across provinces, 257 individuals in Manitoba (5.4%) and 2197 in Ontario (7.5%) died during the index hospitalization. Of those who survived the index hospitalization, 965 in Manitoba (20.3%) and 5301 in Ontario (18.1%) were readmitted to hospital and 399 in Manitoba (8.4%) and 3544 in Ontario (12.1%) died within 1-year of discharge. In both cohorts, individuals in the liver disease subgroup had the highest incidence of both in-hospital and postdischarge mortality (Figure 2).

No violations of the proportional hazards assumption were observed in the unadjusted and multivariable regression models (eFigure 2 in Supplement 1). After adjusting for covariates, individuals in the liver disease subgroup had the highest risk of in-hospital mortality (Ontario: adjusted odds ratio [aOR], 3.80; 95% CI, 2.60-5.53; Manitoba: aOR, 3.12; 95% CI, 1.22-7.96) and postdischarge mortality (Ontario: adjusted hazard ratio [aHR], 3.85; 95% CI, 2.83-5.25; Manitoba: aHR, 2.38; 95% CI, 1.07-5.30) relative to the acute intoxication subgroup (Table 3; eTable 10 in Supplement 1). In Ontario, individuals in the frequent service use subgroups had the highest hazard of alcohol-related hospital readmission relative to the acute intoxication subgroup (hospital-based: aHR, 6.33, 95% CI, 5.09-7.87; all types: aHR, 5.57; 95% CI, 4.47-6.94), followed by individuals in the liver disease subgroup (aHR, 3.15; 95% CI, 2.63-5.70). In Manitoba, individuals in the liver disease subgroup had the highest hazard of alcohol-related hospital readmission relative to the acute intoxication subgroup (aHR, 2.88; 95% CI, 1.75-4.74).

**Table 3.** Results from the Multivariable Regression Models for In-Hospital Mortality, Readmission, and Postdischarge Mortality

Variable	In-hospital mortality		Readmission		Postdischarge mortality	
	OR (95% CI)	aOR (95% CI)	HR (95% CI)	aHR (95% CI)	HR (95% CI)	aHR (95% CI)
Subgroup						
Acute intoxication	1 [Reference]	1 [Reference]	1 [Reference]	1 [Reference]	1 [Reference]	1 [Reference]
Harmful use	1.84 (1.27-2.68)	1.01 (0.69-1.49)	1.08 (0.87-1.33)	1.21 (0.97-1.51)	2.31 (1.69-3.16)	1.22 (0.89-1.67)
Dependence	2.15 (1.50-3.10)	1.21 (0.83-1.76)	1.21 (0.98-1.49)	1.36 (1.10-1.68)	2.40 (1.77-3.27)	1.33 (0.97-1.81)
Withdrawal	1.57 (1.08-2.28)	0.91 (0.62-1.34)	1.84 (1.50-2.27)	2.15 (1.74-2.66)	2.08 (1.52-2.84)	1.18 (0.86-1.62)
Liver disease	8.84 (6.16-12.69)	3.80 (2.60-5.53)	3.27 (2.65-4.03)	3.69 (2.97-4.58)	9.35 (6.90-12.69)	3.85 (2.83-5.25)
Frequent service use (hospital-based)	2.23 (1.49-3.34)	1.24 (0.82-1.89)	7.27 (5.90-8.96)	6.33 (5.09-7.87)	3.92 (2.84-5.43)	1.77 (1.28-2.46)
Frequent service use (all types)	0.82 (0.50-1.35)	0.60 (0.36-1.00)	6.22 (5.02-7.71)	5.57 (4.47-6.94)	2.62 (1.86-3.71)	1.55 (1.10-2.20)
Age (per year)	NA	1.03 (1.02-1.03)	NA	0.99 (0.99-0.99)	NA	1.03 (1.03-1.04)
Sex (Reference, male)	NA	0.89 (0.80-0.99)	NA	1.00 (0.94-1.06)	NA	0.86 (0.79-0.93)
Income quintile						
1 (lowest)	NA	1 [Reference]	NA	1 [Reference]	NA	1 [Reference]
2	NA	0.96 (0.85-1.09)	NA	0.90 (0.84-0.97)	NA	0.92 (0.84-1.01)
3	NA	0.95 (0.83-1.08)	NA	0.83 (0.76-0.90)	NA	0.78 (0.70-0.87)
4	NA	0.82 (0.71-0.96)	NA	0.85 (0.78-0.94)	NA	0.74 (0.66-0.83)
5 (highest)	NA	0.79 (0.68-0.92)	NA	0.87 (0.79-0.96)	NA	0.75 (0.67-0.84)
Statistical area classification						
Large metropolitan	NA	1 [Reference]	NA	1 [Reference]	NA	1 [Reference]
Medium metropolitan	NA	1.13 (0.87-1.46)	NA	0.99 (0.84-1.18)	NA	0.91 (0.84-1.13)
Small metropolitan	NA	1.31 (1.13-1.52)	NA	1.06 (0.96-1.17)	NA	1.13 (1.00-1.28)
Rural (strong MIZ)	NA	1.21 (1.00-1.46)	NA	0.94 (0.82-1.08)	NA	1.15 (1.00-1.34)
Rural (moderate MIZ)	NA	1.05 (0.85-1.29)	NA	0.93 (0.80-1.08)	NA	0.99 (0.84-1.16)
Rural (weak MIZ)	NA	1.01 (0.75-1.37)	NA	1.11 (0.95-1.30)	NA	0.92 (0.71-1.17)
Remote	NA	0.70 (0.41-1.21)	NA	1.19 (0.98-1.46)	NA	0.77 (0.52-1.13)
ADG score (per point)	NA	1.02 (1.02-1.02)	NA	1.01 (1.01-1.02)	NA	1.02 (1.02-1.03)
Psychiatric comorbidity						
None	NA	1 [Reference]	NA	1 [Reference]	NA	1 [Reference]
Outpatient	NA	0.62 (0.53-0.73)	NA	1.23 (1.12-1.34)	NA	0.99 (0.88-1.10)
ED visit	NA	0.75 (0.64-0.89)	NA	1.17 (1.07-1.28)	NA	0.86 (0.76-0.97)
Hospitalization	NA	0.81 (0.72-0.91)	NA	1.04 (0.96-1.12)	NA	0.91 (0.83-0.99)

Abbreviations: ADG, aggregated diagnostic group; aHR, adjusted hazard ratio; aOR, adjusted odds ratio; ED, emergency department; MIZ, metropolitan influenced zone; NA, not applicable.

## Discussion

In this population-based retrospective cohort study, we identified multiple subgroups of individuals who were hospitalized for alcohol-related harms. These subgroups had unique sociodemographic and clinical characteristics, and there were clear differences in the risk of adverse outcomes between certain subgroups. Results highlighted cohorts at particularly high risk, including (1) a small subset of individuals with alcohol-related liver disease (approximately 15% of the cohort) who were at the highest risk of mortality, and (2) a small subset of individuals (approximately 10% of the cohort) who had a history of high-frequency alcohol-related health service use and were at the highest risk of readmission. This provides insight into the diverse subpopulations that are captured by the indicators used to identify alcohol harms in health administrative data. It also suggests that there are small subgroups of individuals who may require prioritization in efforts to reduce the high rates of adverse outcomes among those experiencing alcohol-related hospitalizations.

Research in Canada in past 5 years has highlighted high national rates of alcohol-related health service use, the burden this has on the health care system (with an estimated annual cost of approximately $6 billion^29^), and the high risk of recurrent harm among individuals who experience alcohol-related ED visits and hospitalizations.^2,14,15^ All this research, including corroborating international reports,^1^ has generated substantial interest in monitoring national trends in alcohol-related hospitalizations and developing strategies to reduce the risk of postdischarge harm and recurrent health service use. However, much of this work amalgamates everyone who experiences an alcohol-related hospitalization into a single cohort of individuals with a presumed AUD.^6,30,31,32,33,34^ In turn, there has been relatively little focus on the clinical diversity that exists within this population or if certain subgroups merit prioritization in efforts to reduce the risk of adverse outcomes.

This study illustrated several distinct clinical subgroups within the larger cohort of individuals who are hospitalized for alcohol-related harms. These subgroups spanned the spectrum of clinical presentations related to alcohol use, ranging from those receiving low-frequency care for acute intoxication to those receiving intensive care for severe AUD and liver disease. The risk of adverse outcomes, namely readmission and mortality, differed between these subgroups, with a few small subgroups driving up the high rates of recurrent health service use and mortality seen in the overall cohort. Namely, relative to those in the acute intoxication subgroup, individuals in the high-frequency service use subgroup had approximately 5-fold higher rates of readmission and those in the liver disease subgroup had roughly 10-fold higher rates of both in-hospital and postdischarge mortality. In turn, targeted clinical strategies tailored to the unique needs of these specific high-risk subpopulations may be warranted.^21^

The presence of these clinically distinct subgroups also suggests that future epidemiological work on alcohol-related health service use should consider their presence during both study design and data interpretation. For example, a 2020 US study^35^ found that national rates of alcohol-related hospitalizations increased by 3.5% and national rates of in-hospital mortality decreased by 25% between 1998 and 2016. It was hypothesized that this may be due to improvements in inpatient care for individuals with AUD over time. While this may be true, it would also be important to consider if there have been shifts in the relative distribution of types of hospitalization (eg, an increase in the proportion of hospitalizations for acute intoxication vs liver disease) that might underlie the overall changes in in-hospital mortality. Similar considerations should also be made when interpreting the results of other recent population-based analyses of alcohol-related health service use in Canada^4,6,7,31,32^ and internationally.^34,35,36^ Of course, there will be some variation in the subtypes of individuals who experience alcohol-related hospitalizations between jurisdictions. However, the fact that we observed nearly identical subgroups in 2 independent Canadian populations with different sociodemographic compositions, alcohol regulation strategies, and health care systems supports the generalizability of our findings between Canadian and international contexts.

### Limitations

This study had several limitations. First, both Manitoba and Ontario have universal health insurance plans that provide residents with free access to medical services. In turn, while the external validity of the study findings is strengthened by analogous results obtained in both provinces, there may be differences in how people access health services for alcohol-related harms in Canada vs jurisdictions without universal health care. Furthermore, there were 2 subgroups observed in Ontario that were not observed in Manitoba. This may be partially related to differences in statistical power afforded by the larger sample size in Ontario but may also reflect variability in the subgroups of individuals who experience alcohol-related hospitalization between jurisdictions. This regional variability should be considered in studies seeking to replicate these findings in an international context. Second, due to data availability, this study did not consider prior use of AUD medications (eg, naltrexone, acamprosate) or private addiction services when characterizing prior alcohol-related health service use. With regards to the former, however, previous Canadian data indicates that fewer than 2% of individuals with AUD receive these medications,^31,32^ which may reduce the impact of this limitation.

Finally, this study used data from 2015-2019. There were shifts in the frequency and type of alcohol-related health service use during the COVID-19 pandemic in Canada,^6,22^ which may have changed the nature and distribution of the subgroups of individuals who experience alcohol-related hospitalizations. In part, the use of data from prior to the pandemic helped to mitigate any bias introduced by these potentially transient shifts in health service use; however, additional work will be required to identify whether there have been lasting changes to the pattern of alcohol-related health service use in the postpandemic era.

## Conclusion

This study identified distinct clinical subgroups of individuals hospitalized for alcohol-related harms. These subgroups followed a severity gradient, and those in the more severe categories accounted for most of the adverse in-hospital and postdischarge outcomes. Future epidemiological research on alcohol-related health service use should consider the presence of these subgroups in their study design. Similarly, efforts to reduce high rates of readmission and mortality among individuals experiencing alcohol-related hospitalizations may consider prioritizing those at the highest risk of short-term harm, including individuals with alcohol-related liver disease and high frequency health service use.

## Notes

Supplement 1. **eTable 1.** Databases Used to Create Cohorts and Variables **eTable 2.** Ontario Cohort Creation: Tabulation of Individuals Excluded From the Cohort by Reason **eTable 3.** LCA Model Fit With Increasing Class Numbers **eTable 4.** Model Fit for Manitoba LCA: Average of Posterior Probabilities of Class Membership by Assigned Class **eTable 5.** Model Fit for Ontario LCA: Average of Posterior Probabilities of Class Membership by Assigned Class **eTable 6. **Diagnoses and Prior Alcohol-Related Health Service Use Across the Five Classes Identified in Manitoba **eTable 7.** Demographic and Clinical Characteristics of the Manitoba Cohort, Stratified by Subgroup **eTable 8. **One-Year Incidence of Study Outcomes in Manitoba, Stratified by Subgroup **eTable 9. **One-Year Incidence of Study Outcomes in Ontario, Stratified by Subgroup **eTable 10. **Results From the Multivariable Regression Models for In-Hospital Mortality, Readmission, and Postdischarge Mortality in the Manitoba Cohort **eFigure 1.** Schematic of Data Collection Windows **eFigure 2. **Kaplan-Meier (KM) Curves for Readmission and Mortality Following the Index Alcohol-Related Hospitalization in Ontario, Stratified by Subgroup **eAppendix.** STROBE Statement—Checklist of Items That Should Be Included in Reports of Cohort Studies

Supplement 2.
Data Sharing Statement

## References

[1] Griswold MG, Fullman N, Hawley C, ; GBD 2016 Alcohol Collaborators. Alcohol use and burden for 195 countries and territories, 1990-2016: a systematic analysis for the Global Burden of Disease Study 2016. Lancet. 2018;392(10152):1015-1035. doi:10.1016/S0140-6736(18)31310-2 30146330 PMC6148333

[2] Sherk A. At-a-glance—the alcohol deficit: Canadian government revenue and societal costs from alcohol. Health Promot Chronic Dis Prev Can. 2020;40(5-6):139-142. 32529973 10.24095/hpcdp.40.5/6.02

[3] Canadian Institute for Health Information. Alcohol Harm in Canada: Examining Hospitalizations Entirely Caused by Alcohol and Strategies to Reduce Alcohol Harm. 2017. Accessed August 17, 2023. https://www.cihi.ca/sites/default/files/document/report-alcohol-hospitalizations-en-web.pdf

[4] Nickel NC, Bolton J, MacWilliam L, . Health and Social Outcomes Associated with High-Risk Alcohol Use. Manitoba Centre for Health Policy; 2018.

[5] Myran DT, Hsu AT, Smith G, Tanuseputro P. Rates of emergency department visits attributable to alcohol use in Ontario from 2003 to 2016: a retrospective population-level study. CMAJ. 2019;191(29):E804-E810. doi:10.1503/cmaj.181575 31332048 PMC6645924

[6] Myran D, Friesen EL, Pugliese M, . Changes in health service use due to alcohol during the COVID-19 pandemic among individuals with and individuals without pre-existing alcohol-related medical diagnoses. Can J Public Health. 2023;114(2):185-194. doi:10.17269/s41997-023-00739-8 36719599 PMC9888341

[7] Friesen EL, Yu W, Buajitti E, Selby P, Rosella L, Kurdyak P. Clarifying rural-urban disparities in alcohol-related emergency department visits and hospitalizations in Ontario, Canada: a spatial analysis. J Rural Health. 2023;39(1):223-232. doi:10.1111/jrh.12702 35866637

[8] Friesen EL, Myran D, Yu W, Rosella L, Selby P, Kurdyak P. Rural-urban disparities in post-discharge outcomes following alcohol-related hospitalizations in Ontario, Canada: A retrospective cohort study. Drug Alcohol Depend. 2022;238:109568. doi:10.1016/j.drugalcdep.2022.109568 35850027

[9] Bergman D, Hagström H, Capusan AJ, . Incidence of ICD-based diagnoses of alcohol-related disorders and diseases from Swedish nationwide registers and suggestions for coding. Clin Epidemiol. 2020;12:1433-1442. doi:10.2147/CLEP.S285936 33408530 PMC7781026

[10] Rehm J. The risks associated with alcohol use and alcoholism. Alcohol Res Health. 2011;34(2):135-143. 22330211 PMC3307043

[11] Canadian Institute for Health Information. Hospitalizations Entirely Caused by Alcohol — Appendices to Indicator Library, May 2020. Updated November 2020. Accessed August 17, 2023. https://www.cihi.ca/en/indicators/hospitalizations-entirely-caused-by-alcohol

[12] National Center for Chronic Disease Prevention and Health Promotion. Alcohol-Related ICD Codes. Centers for Disease Control and Prevention webpage. Accessed July 4, 2023. https://www.cdc.gov/alcohol/ardi/alcohol-related-icd-codes.html

[13] Bartoli F, Carrà G, Crocamo C, Clerici M. From DSM-IV to DSM-5 alcohol use disorder: an overview of epidemiological data. Addict Behav. 2015;41:46-50. doi:10.1016/j.addbeh.2014.09.029 25305657

[14] Friesen EL, Yu W, Kurdyak P. Outpatient psychiatric service use is associated with a reduced risk of 1-year readmission and mortality following alcohol-related hospitalizations: A historical cohort study. Acta Psychiatr Scand. 2023;148(2):179-189. doi:10.1111/acps.13560 37221899

[15] Hulme J, Sheikh H, Xie E, Gatov E, Nagamuthu C, Kurdyak P. Mortality among patients with frequent emergency department use for alcohol-related reasons in Ontario: a population-based cohort study. CMAJ. 2020;192(47):E1522-E1531. doi:10.1503/cmaj.191730 33229348 PMC7721258

[16] Statistics Canada. Population Growth in Canada’s Rural Areas, 2016 to 2021. February 9, 2022. Accessed August 17, 2023. https://www12.statcan.gc.ca/census-recensement/2021/as-sa/98-200-x/2021002/98-200-x2021002-eng.cfm

[17] Statistics Canada. Population and Demography Statistics. Accessed August 17, 2023. https://www.statcan.gc.ca/en/subjects-start/population_and_demography

[18] Giesbrecht N, Wettlaufer A, Thomas G, . Pricing of alcohol in Canada: a comparison of provincial policies and harm-reduction opportunities. Drug Alcohol Rev. 2016;35(3):289-297. doi:10.1111/dar.12338 26530717

[19] Marchildon GP, Allin S, Merkur S. Canada: health system review. Health Syst Transit. 2020;22(3):1-194. 33527903

[20] Allin S. Does equity in healthcare use vary across Canadian provinces? Healthcare Policy. 2008;3(4):83-99. doi:10.12927/hcpol.2008.19924 19377331 PMC2645154

[21] Carvalho AF, Heilig M, Perez A, Probst C, Rehm J. Alcohol use disorders. Lancet. 2019;394(10200):781-792. doi:10.1016/S0140-6736(19)31775-1 31478502

[22] Myran DT, Cantor N, Pugliese M, . Sociodemographic changes in emergency department visits due to alcohol during COVID-19. Drug Alcohol Depend. 2021;226:108877. doi:10.1016/j.drugalcdep.2021.108877 34256266 PMC9759020

[23] Myran D, Hsu A, Kunkel E, Rhodes E, Imsirovic H, Tanuseputro P. Socioeconomic and geographic disparities in emergency department visits due to alcohol in Ontario: a retrospective population-level study from 2003 to 2017. Can J Psychiatry. 2022;67(7):534-543. doi:10.1177/07067437211027321 34254563 PMC9234901

[24] Statistics Canada. Standard Geographical Classification (SGC) 2016—Introduction. Statistics Canada. Accessed September 13, 2021. https://www.statcan.gc.ca/eng/subjects/standard/sgc/2016/introduction

[25] Klaassen Z, Wallis CJD, Goldberg H, . The impact of psychiatric utilisation prior to cancer diagnosis on survival of solid organ malignancies. Br J Cancer. 2019;120(8):840-847. doi:10.1038/s41416-019-0390-0 30837680 PMC6474265

[26] Hastings SN, Whitson HE, Sloane R, Landerman LR, Horney C, Johnson KS. Using the past to predict the future: latent class analysis of patterns of health service use of older adults in the emergency department. J Am Geriatr Soc. 2014;62(4):711-715. doi:10.1111/jgs.12746 24635112 PMC3989455

[27] Lythgoe DT, Garcia-Fiñana M, Cox TF. Latent class modeling with a time-to-event distal outcome: a comparison of one, two and three-step approaches. Struct Equ Modeling. 2019;26(1):51-65. doi:10.1080/10705511.2018.1495081

[28] Lergenmuller S, Rueegg CS, Perrier F, . Lifetime sunburn trajectories and associated risks of cutaneous melanoma and squamous cell carcinoma among a cohort of Norwegian women. JAMA Dermatol. 2022;158(12):1367-1377. doi:10.1001/jamadermatol.2022.4053 36197657 PMC9535508

[29] Canadian Institute for Health Information. Your health system: hospitalizations entirely caused by alcohol. Accessed November 24, 2021. https://www.cihi.ca/en/indicators/hospitalizations-entirely-caused-by-alcohol

[30] Huỳnh C, Kisely S, Rochette L, . Measuring substance-related disorders using Canadian Administrative Health Databanks: interprovincial comparisons of recorded diagnostic rates, incidence proportions and mortality rate ratios. Can J Psychiatry. 2022;67(2):117-129. doi:10.1177/07067437211043446 34569874 PMC8978214

[31] Konrad G, Leong C, Bolton JM, . Use of pharmacotherapy for alcohol use disorder in Manitoba, Canada: a whole-population cohort study. PLoS One. 2021;16(9):e0257025. doi:10.1371/journal.pone.0257025 34478448 PMC8415582

[32] Spithoff S, Turner S, Gomes T, Martins D, Singh S. First-line medications for alcohol use disorders among public drug plan beneficiaries in Ontario. Can Fam Physician. 2017;63(5):e277-e283. 28500210 PMC5429069

[33] Kim HM, Smith EG, Stano CM, . Validation of key behaviourally based mental health diagnoses in administrative data: suicide attempt, alcohol abuse, illicit drug abuse and tobacco use. BMC Health Serv Res. 2012;12(1):18. doi:10.1186/1472-6963-12-18 22270080 PMC3280157

[34] Bernstein EY, Baggett TP, Trivedi S, Herzig SJ, Anderson TS. Pharmacologic treatment initiation among Medicare beneficiaries hospitalized with alcohol use disorder. Ann Intern Med. 2023;176(8):1137-1139. doi:10.7326/M23-0641 37364264 PMC10910351

[35] Singh JA, Cleveland JD. Trends in hospitalizations for alcohol use disorder in the US from 1998 to 2016. JAMA Netw Open. 2020;3(9):e2016580. doi:10.1001/jamanetworkopen.2020.16580 32955569 PMC12550831

[36] Sacco P, Unick GJ, Kuerbis A, Koru AG, Moore AA. Alcohol-related diagnoses in hospital admissions for all causes among middle-aged and older adults: trends and cohort differences from 1993 to 2010. J Aging Health. 2015;27(8):1358-1374. doi:10.1177/0898264315583052 25903980 PMC4755348

