# Supplementary material for: Outcomes in Clinical Subgroups of Patients With Alcohol-Related Hospitalizations
Source: JAMA Netw Open. 2024 Jan 31;7(1):e2353971. doi: 10.1001/jamanetworkopen.2023.53971 (PMC10831574; doi:10.1001/jamanetworkopen.2023.53971)
Supplement: Supplement 1. — eTable 1. Databases Used to Create Cohorts and Variables eTable 2. Ontario Cohort Creation: Tabulation of Individuals Excluded From the Cohort by Reason eTable 3. LCA Model Fit With Increasing Class Numbers eTable 4. Model Fit for Manitoba LCA: Average of Posterior Probabilities of Class Membership by Assigned Class eTable 5. Model Fit for Ontario LCA: Average of Posterior Probabilities of Class Membership by Assigned Class eTable 6. Diagnoses and Prior Alcohol-Related Health Service Use Across the Five Classes Identified in Manitoba eTable 7. Demographic and Clinical Characteristics of the Manitoba Cohort, Stratified by Subgroup eTable 8. One-Year Incidence of Study Outcomes in Manitoba, Stratified by Subgroup eTable 9. One-Year Incidence of Study Outcomes in Ontario, Stratified by Subgroup eTable 10. Results From the Multivariable Regression Models for In-Hospital Mortality, Readmission, and Postdischarge Mortality in the Manitoba Cohort eFigure 1. Schematic of Data Collection Windows eFigure 2. Kaplan-Meier (KM) Curves for Readmission and Mortality Following the Index Alcohol-Related Hospitalization in Ontario, Stratified by Subgroup eAppendix. STROBE Statement—Checklist of Items That Should Be Included in Reports of Cohort Studies [file jamanetwopen-e2353971-s001.pdf]

Supplemental Online Content

Friesen EL, Mataruga A, Nickel N, Kurdyak P, Bolton JM. Outcomes in clinical subgroups of patients with alcohol-related hospitalizations. *JAMA Netw Open.* 2024;7(1):e2353971. doi:10.1001/jamanetworkopen.2023.53971

eTable 1. Databases Used to Create Cohorts and Variables

eTable 2. Ontario Cohort Creation: Tabulation of Individuals Excluded From the Cohort by Reason

eTable 3. LCA Model Fit With Increasing Class Numbers

eTable 4. Model Fit for Manitoba LCA: Average of Posterior Probabilities of Class Membership by Assigned Class

eTable 5. Model Fit for Ontario LCA: Average of Posterior Probabilities of Class Membership by Assigned Class

eTable 6. Diagnoses and Prior Alcohol-Related Health Service Use Across the Five Classes Identified in Manitoba

eTable 7. Demographic and Clinical Characteristics of the Manitoba Cohort, Stratified by Subgroup

eTable 8. One-Year Incidence of Study Outcomes in Manitoba, Stratified by Subgroup

eTable 9. One-Year Incidence of Study Outcomes in Ontario, Stratified by Subgroup

eTable 10. Results From the Multivariable Regression Models for In-Hospital Mortality, Readmission, and Postdischarge Mortality in the Manitoba Cohort

eFigure 1. Schematic of Data Collection Windows

eFigure 2. Kaplan-Meier (KM) Curves for Readmission and Mortality Following the Index Alcohol-Related Hospitalization in Ontario, Stratified by Subgroup

eAppendix. STROBE Statement—Checklist of Items That Should Be Included in Reports of Cohort Studies

This supplemental material has been provided by the authors to give readers additional information about their work.

© 2024 Friesen EL et al. *JAMA Network Open.*

eTable 1. Databases Used to Create Cohorts and Variables

Category	Variable	Ontario Database(s)	Manitoba Database(s)
Exposures	Index hospitalization code(s)	Discharge Abstract Database (DAD)	Hospital Abstracts
	Count of previous alcohol-related outpatient visits*	OHIP Claims	Medical Claims/Services
	Count of previous alcohol-related ED visits	National Ambulatory Care Reporting System (NACRS)	Emergency Department Information System (EDIS)
	Count of previous alcohol-related hospitalizations	DAD	Hospital Abstracts
Outcomes	In-hospital Mortality	Registered Persons Database	Manitoba Health Insurance Registry
	Alcohol-related hospital readmissions	DAD	Hospital Abstracts
	All-cause mortality (post-discharge)	Registered Persons Database	Manitoba Health Insurance Registry
Covariates	Age	Registered Persons Database	Manitoba Health Insurance Registry
	Sex	Registered Persons Database	Manitoba Health Insurance Registry
	Income Quintile	Registered Persons Database, Postal Code Conversion File	Manitoba Health Insurance Registry, Postal Code Conversion File
	Statistical Area Classification (Rurality)	Registered Persons Database, Postal Code Conversion File	Manitoba Health Insurance Registry, Postal Code Conversion File
	Psychiatric Comorbidity	DAD, OMHRS, NACRS, OHIP Claims	Hospital Abstracts, EDIS, Medical Claims/Services
	Medical comorbidity (Johns Hopkins ADG Score)	DAD, SDS, NACRS, OHIP Claims	Hospital Abstracts, Medical Claims/Services

OMHRS: Ontario Mental Health Reporting System
SDS: Same-Day Surgery Database
* includes all publicly funded outpatient addiction services that involved a physician billing the province for services rendered (e.g., Rapid Access Addiction Medicine clinics)

© 2024 Friesen EL et al. *JAMA Network Open.*

eTable 2. Ontario Cohort Creation: Tabulation of Individuals Excluded From the Cohort by Reason

Reason for Exclusion	Ontario	Manitoba
Records Identified	55,736	1,039,732
Wrong timeframe (before Jan 1, 2017 or after Dec 31, 2018)	NA	608,960
Non-alcohol-related hospitalization	NA	418,710
Invalid identifier (Ontario = ICES Key Number, Manitoba: scrambler personal health identification number)	21	NA
Out of Province	1,347	4,826
Invalid death date	41	NA
Not eligible for universal healthcare insurance for the whole study timeframe	1,776	7
Age under 10 or over 105	21	0
IKN had more than 1 index event, but this one not being randomly picked	17,478	2,476
Included for analysis	35,052	4,753

Note: there were different workflows from collecting and cleaning the data between provinces, which translate into some differences in the exclusion workflow described above. For example, the initial data cut in Ontario excluded individuals with non-alcohol-related hospitalizations or those in the wrong timeframe. Similarly, the initial data cut in Manitoba excluded individuals with invalid death dates or identifiers. This has been illustrated with 'NA's.

© 2024 Friesen EL et al. *JAMA Network Open.*

eTable 3. LCA Model Fit With Increasing Class Numbers

	Manitoba		Ontario	
**Classes**	**AIC**	**BIC**	**AIC**	**BIC**
2	51,003	51,501	364,191	364,945
3	50,264	51,014	356,858	357,993
4	49,591	50,593	352,990	354,506
5	48,162	49,417	348,626	350,523
6	47,910	49,418	346,527	348,806
7	47,804	49,564	340,073	342,732
8	47,159	49,170	339,134	342,174

AIC: Akaike Information Criterion
BIC: Bayesian Information Criterion

© 2024 Friesen EL et al. *JAMA Network Open.*

eTable 4. Model Fit for Manitoba LCA: Average of Posterior Probabilities of Class Membership by Assigned Class

	Average Posterior Probability of Class Membership				
**Assigned Class**	Harmful use	Liver Disease	Withdrawal	Alcohol Dependence	Acute Intoxication
Harmful use	0.99	<0.01	<0.01	<0.01	<0.01
Liver Disease	<0.01	0.99	<0.01	<0.01	<0.01
Withdrawal	<0.01	<0.01	0.99	<0.01	<0.01
Alcohol Dependence	0.01	0.01	<0.01	0.98	<0.01
Acute Intoxication	0.01	<0.01	<0.01	0.01	0.98

© 2024 Friesen EL et al. *JAMA Network Open.*

eTable 5. Model Fit for Ontario LCA: Average of Posterior Probabilities of Class Membership by Assigned Class

	Average Posterior Probability of Class Membership						
**Assigned Class**	Frequent Use - All Types	Liver Disease	Alcohol Dependence	Acute Intoxication	Frequent Use - Hospital-Based	Withdrawal	Harmful Use
Frequent Use - All Types	0.81	0.01	0.02	<0.01	0.09	0.04	0.02
Liver Disease	<0.01	0.95	0.01	<0.01	0.02	0.02	0.01
Alcohol Dependence	<0.01	<0.01	0.99	<0.01	<0.01	<0.01	<0.01
Acute Intoxication	0.01	<0.01	<0.01	0.99	<0.01	<0.01	<0.01
Frequent Use - Hospital-Based	0.02	0.03	<0.01	<0.01	0.93	<0.01	0.03
Withdrawal	<0.01	<0.01	<0.01	<0.01	<0.01	0.99	<0.01
Harmful Use	<0.01	<0.01	0.01	<0.01	<0.01	<0.01	0.98

© 2024 Friesen EL et al. *JAMA Network Open.*

eTable 6. Diagnoses and Prior Alcohol-Related Health Service Use Across the Five Classes Identified in Manitoba

Previous Service Use	Count	Acute Intoxication*	Harmful Use*	Alcohol Dependence*	Withdrawal*	Liver Disease*
Alcohol-related Outpatient Visits (%)	0	79.84	79.68	75.13	73.32	54.14
	1	11.63	12.75	14.74	15.80	20.53
	2+	8.53	7.56	10.13	10.88	25.31
Alcohol-related ED visits (%)	0	87.60	93.30	91.25	88.43	87.61
	1	8.53	4.90	5.00	6.56	7.61
	2+	3.88	1.80	3.75	5.01	4.78
Alcohol-related Hospitalizations (%)	0	82.17	86.02	79.34	70.90	50.44
	1	6.98	7.20	9.21	11.92	15.04
	2+	10.85	6.77	11.45	17.18	34.51
**ICD-10 Code**	**Description**					
F100	Alcohol intoxication	4.65	<1.00	13.75	1.38	<1.00
F101	Harmful alcohol use	10.08	100.00	0.00	6.04	9.73
F102	Alcohol dependence	3.88	0.00	37.17	4.58	4.60
F103	Alcohol withdrawal	6.20	<1.00	<1.00	100.00	7.61
F104	Alcohol withdrawal with delirium	<1.00	<1.00	7.57	0.00	<1.00
F105	Alcohol-related psychotic disorder	<1.00	<1.00	2.96	<1.00	0.00
F106	Alcohol-related amnesic syndrome	0.00	<1.00	2.63	<1.00	<1.00
F107	Alcohol-related residual psychotic disorder	0.00	0.00	2.04	<1.00	<1.00
F108	Alcohol-related other mental and behavioral disorder	<1.00	<1.00	<1.00	0.00	0.00
F109	Alcohol-related unspecified mental and behavioral disorder	<1.00	<1.00	9.47	<1.00	<1.00
T510	Toxic effect of ethanol	77.52	0.00	0.00	0.00	0.00
T511	Toxic effect of methanol	6.98	0.00	0.00	0.00	0.00
T512	Toxic effect of isopropyl alcohol	3.88	0.00	0.00	0.00	0.00
K700	Alcoholic liver disease	0.00	<1.00	<1.00	<1.00	<1.00
K701	Alcoholic fatty liver	<1.00	1.15	3.68	2.42	4.78
K702	Alcoholic hepatitis	0.00	0.00	<1.00	0.00	0.00

© 2024 Friesen EL et al. *JAMA Network Open.*

K703	Alcoholic liver cirrhosis	0.00	0.00	0.00	0.00	100.00
K704	Alcoholic hepatic failure	0.00	<1.00	3.88	<1.00	9.73
K709	Alcoholic liver disease unspecified	0.00	<1.00	1.32	<1.00	<1.00
G312	Degeneration of nervous system due to alcohol	0.00	<1.00	1.12	<1.00	3.89
G621	Alcoholic polyneuropathy	0.00	0.00	<1.00	<1.00	0.00
G721	Alcoholic myopathy	0.00	0.00	<1.00	0.00	0.00
I426	Alcoholic cardiomyopathy	0.00	<1.00	1.05	<1.00	<1.00
K292	Alcoholic gastritis	0.00	<1.00	1.97	<1.00	<1.00
K852	Alcohol-induced acute pancreatitis	0.00	1.66	7.76	2.16	<1.00
K860	Alcohol-induced chronic pancreatitis	0.00	<1.00	2.04	<1.00	<1.00
O35401	Maternal care for suspected damage to fetus from alcohol use	0.00	<1.00	<1.00	0.00	0.00
Q860	Fetal alcohol spectrum disorders	0.00	<1.00	6.51	0.00	0.00
X45	Accidental alcohol poisoning	43.41	<1.00	0.00	<1.00	0.00
X65	Intentional alcohol poisoning	38.76	0.00	0.00	<1.00	0.00
Y15	Alcohol poisoning undetermined intent	13.18	0.00	0.00	0.00	0.00

For clarity, shading is representative of the proportion of each subgroup with that characteristic (darker shade, higher proportion)
* Numbers represent the % of each subgroup with that characteristic. Columns do not sum to 100% because individuals can have multiple diagnoses associated with the index hospitalization.

© 2024 Friesen EL et al. *JAMA Network Open.*

eTable 7. Demographic and Clinical Characteristics of the Manitoba Cohort, Stratified by Subgroup

Characteristic	Acute Intoxication n = 129	Harmful use n = 1,387	Alcohol Dependence n = 1,517	Withdrawal n = 1,157	Liver Disease n = 563	Overall n = 4,753	*p*
Age (years)	39.9 (15.9)	45.9 (18.5)	48.4 (18.3)	49.4 (15.2)	59.2 (11.7)	48.9 (17.5)	<0.01
% Female	50.39	43.33	37.84	29.9	35.52	37.58	<0.01
Income Quintile							
1 (lowest)	41.86	43.26	41.13	38.12	32.68	40.04	<0.01
2	21.72	20.26	22.81	20.57	22.38	21.44	
3	12.40	12.83	12.99	15.73	15.10	13.84	
4	10.08	12.69	11.60	14.00	17.23	13.13	
5 (highest)	13.95	10.96	11.47	11.58	12.61	11.55	
Statistical Area Classification							
Large Metropolitan	52.71	46.94	54.19	49.70	67.32	52.49	<0.01
Small Metropolitan	12.40	14.92	8.04	14.26	6.93	11.55	
Rural (strong MIZ)	3.10	2.60	3.82	3.89	4.62	3.56	
Rural (moderate MIZ)	6.20	7.79	9.76	9.33	9.24	8.92	
Rural (weak MIZ)	20.16	19.75	18.13	17.80	9.06	17.50	
Remote	5.43	8.00	6.06	5.01	2.84	5.98	
ADG Score	5.07 (3.47)	5.22 (3.61)	5.42 (3.62)	5.09 (3.49)	7.46 (3.94)	5.51 (3.69)	<0.01
Psychiatric Comorbidity							
None	31.01	48.38	52.54	51.94	65.72	52.16	<0.01
Outpatient	53.49	34.90	35.33	40.88	30.73	36.50	
ED visit	4.65	2.24	2.70	3.11	1.42	2.57	
Hospitalization	10.85	14.49	9.43	4.06	2.13	8.77	

ADG: Aggregated Diagnostic Group
* significant differences gauged using χ^2 tests for independence (categorical) and one-way analysis of variance (continuous)

© 2024 Friesen EL et al. *JAMA Network Open.*

eTable 8. One-Year Incidence of Study Outcomes in Manitoba, Stratified by Subgroup

Outcome	Acute Intoxication	Harmful Use	Dependence	Withdrawal	Liver Disease	Overall
% Died in Hospital	3.88	3.32	4.42	1.99	20.78	5.43
% Readmission within 1-year	13.71	14.09	17.03	25.13	39.24	20.31
% Died within 1-year	4.84	5.52	7.45	6.70	25.56	8.41

© 2024 Friesen EL et al. *JAMA Network Open.*

eTable 9. One-Year Incidence of Study Outcomes in Ontario, Stratified by Subgroup

Outcome	Acute Intoxication	Harmful Use	Alcohol Dependence	Withdrawal	Liver Disease	Frequent Use - All Types	Frequent Use - Hospital-Based	Overall
% Died in Hospital	2.84	5.21	6.07	4.39	20.76	2.36	6.55	7.50
% Readmission within 1-year	9.34	9.77	11.12	16.49	26.74	46.04	50.53	18.06
% Died within 1-year	4.09	9.08	9.43	8.19	31.30	10.54	14.22	12.06

© 2024 Friesen EL et al. *JAMA Network Open.*

eTable 10. Results From the Multivariable Regression Models for In-Hospital Mortality, Readmission, and Postdischarge Mortality in the Manitoba Cohort

	In-Hospital Mortality				Readmission				Post-discharge Mortality			
**Variable**	**OR**	**95% CI**	**aOR**	**95% CI**	**HR**	**95% CI**	**aHR**	**95% CI**	**HR**	**95% CI**	**aHR**	**95% CI**
Subgroup												
Acute Intoxication	REF	REF	REF	REF	REF	REF	REF	REF	REF			
Harmful Use	0.86	0.35 - 2.25	0.62	0.24 - 1.61	1.05	0.65 - 1.69	1.01	0.62 - 1.63	1.1	0.49 - 2.45	0.8	0.36 - 1.80
Alcohol Dependence	1.17	0.47 - 2.92	0.78	0.30 - 1.99	1.28	0.80 - 2.06	1.23	0.76 - 1.98	1.5	0.68 - 3.30	0.98	0.44 - 2.17
Withdrawal	0.53	0.20 - 1.41	0.35	0.12 - 0.89	1.95	1.22 - 3.13	1.96	1.21 - 3.15	1.35	0.61 - 3.02	0.92	0.41 - 2.05
Liver Disease	6.62	2.67 - 16.40	3.12	1.22 - 7.96	3.47	2.14 - 5.62	2.88	1.75 - 4.74	5.75	2.61 - 12.68	2.38	1.07 - 5.30
Age (per year)			1.04	1.03 - 1.05			0.99	0.99 - 1.00			1.03	1.03 - 1.04
Sex (Male Ref.)			0.93	0.70 - 1.23			1.11	0.96 - 1.27			1.08	0.86 - 1.34
Income Quintile												
1 (lowest)			REF	REF			REF	REF			REF	REF
2			0.86	0.60 - 1.25			1.01	0.85 - 1.20			0.99	0.76 - 1.30
3			1.11	0.74 - 1.66			0.90	0.73 - 1.12			0.90	0.65 - 1.24
4			0.81	0.52 - 1.24			0.85	0.68 - 1.07			0.73	0.51 - 1.03
5 (highest)			0.98	0.62 - 1.53			0.95	0.76 - 1.20			0.72	0.50 - 1.05
Statistical Area Classification												
Large Metropolitan			REF	REF			REF	REF			REF	REF
Small Metropolitan			1.67	1.09 - 2.56			1.27	1.05 - 1.53			0.83	0.55 - 1.25
Rural (strong MIZ)			0.75	0.35 - 1.58			1.15	0.83 - 1.59			1.23	0.75 - 2.04
Rural (moderate MIZ)			1.02	0.65 - 1.61			1.17	0.95 - 1.44			1.09	0.78 - 1.54
Rural (weak MIZ)			0.78	0.50 - 1.23			1.05	0.88 - 1.24			0.84	0.61 - 1.16
Remote			0.94	0.47 - 1.87			1.05	0.81 - 1.37			1.06	0.66 - 1.70
ADG Score (per point)			1.03	0.99 - 1.06			1.12	1.09 - 1.14			1.09	1.06 - 1.12
Psychiatric Comorbidity												
None			REF	REF			REF	REF			REF	REF
Outpatient			0.85	0.62 - 1.15			0.90	0.76 - 1.05			0.80	0.63 - 1.00
ED visit			0.37	0.09 - 1.55			1.34	0.94 - 1.92			0.37	0.14 - 0.99
Hospitalization			0.52	0.24 - 1.10			1.09	0.85 - 1.40			0.35	0.18 - 0.66

ADG: Aggregated Diagnostic Group
OR: odds ratio
HR: hazard ratio
CI: confidence interval

© 2024 Friesen EL et al. *JAMA Network Open.*

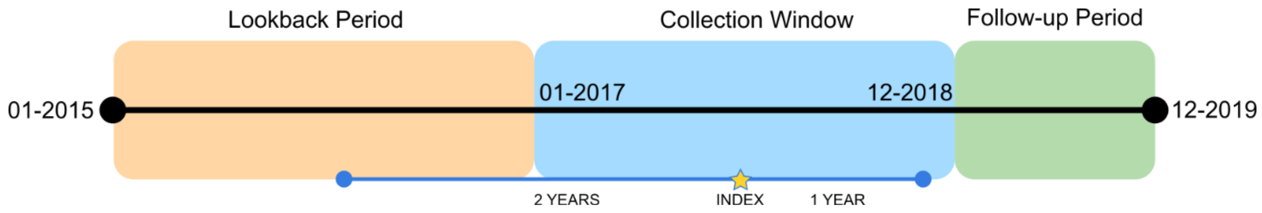

eFigure 1. Schematic of Data Collection Windows. The blue bar on the bottom represents a theoretical member of the cohort, with (1) an index hospitalization between January 1, 2017, and December 31, 2018 (yellow star), (2) a 2-year look back period for prior alcohol-related health service use, medical comorbidities, and psychiatric comorbidities, and (3) a 1-year follow-up to identify readmission and mortality. The colored panes indicate the theoretical maximums of each collection window, whereby the index hospitalization could occur between January 1, 2017 and December 31, 2018 such that the maximum 2-year lookback would be to January 1, 2015 and the maximum 1-year follow-up would be to December 31, 2019.

© 2024 Friesen EL et al. *JAMA Network Open.*

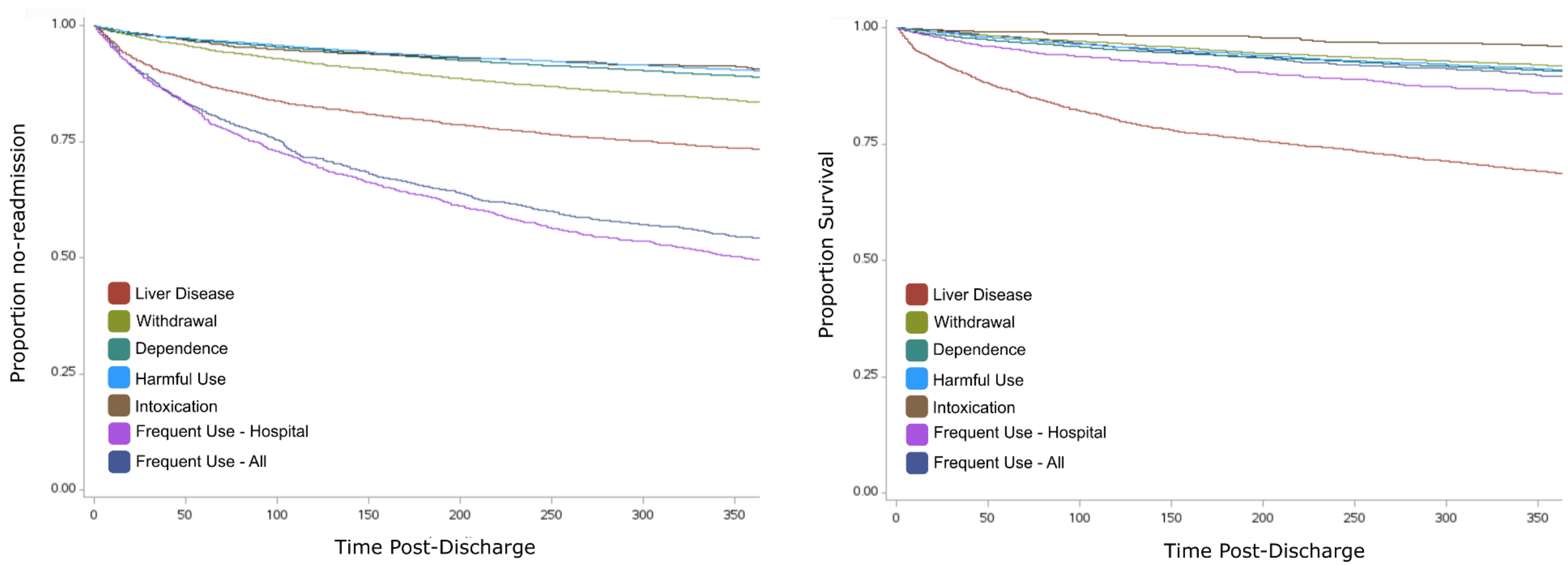

eFigure 2. Kaplan-Meier (KM) Curves for Readmission and Mortality Following the Index Alcohol-Related Hospitalization in Ontario, Stratified by Subgroup. The purpose of these curves was to evaluate for violations of the proportional hazards assumption, which would manifest as a crossing or divergence of lines. We do not observe this, indicating that the assumption is not violated in the models presented in Table 3 in the main text.

© 2024 Friesen EL et al. *JAMA Network Open.*

eAppendix. STROBE Statement—Checklist of Items That Should Be Included in Reports of Cohort Studies

	Item No	Recommendation	Page No
**Title and abstract**	1	(*a*) Indicate the study's design with a commonly used term in the title or the abstract<br>(*b*) Provide in the abstract an informative and balanced summary of what was done and what was found	1-3
**Introduction**			
Background/rationale	2	Explain the scientific background and rationale for the investigation being reported	5
Objectives	3	State specific objectives, including any prespecified hypotheses	6
**Methods**			
Study design	4	Present key elements of study design early in the paper	6
Setting	5	Describe the setting, locations, and relevant dates, including periods of recruitment, exposure, follow-up, and data collection	6-7
Participants	6	(*a*) Give the eligibility criteria, and the sources and methods of selection of participants. Describe methods of follow-up<br>(*b*) For matched studies, give matching criteria and number of exposed and unexposed	6-7, sTable 1 & 2
Variables	7	Clearly define all outcomes, exposures, predictors, potential confounders, and effect modifiers. Give diagnostic criteria, if applicable	8-9
Data sources/ measurement	8*	For each variable of interest, give sources of data and details of methods of assessment (measurement). Describe comparability of assessment methods if there is more than one group	sTable 1
Bias	9	Describe any efforts to address potential sources of bias	10
Study size	10	Explain how the study size was arrived at	NA
Quantitative variables	11	Explain how quantitative variables were handled in the analyses. If applicable, describe which groupings were chosen and why	8-10
Statistical methods	12	(*a*) Describe all statistical methods, including those used to control for confounding<br>(*b*) Describe any methods used to examine subgroups and interactions<br>(*c*) Explain how missing data were addressed<br>(*d*) If applicable, explain how loss to follow-up was addressed<br>(*e*) Describe any sensitivity analyses	9-11
**Results**			
Participants	13*	(a) Report numbers of individuals at each stage of study—eg numbers potentially eligible, examined for eligibility, confirmed eligible, included in the study, completing follow-up, and analysed<br>(b) Give reasons for non-participation at each stage<br>(c) Consider use of a flow diagram	11, sTable 2
Descriptive data	14*	(a) Give characteristics of study participants (eg demographic, clinical, social) and information on exposures and potential confounders<br>(b) Indicate number of participants with missing data for each variable of interest<br>(c) Summarise follow-up time (eg, average and total amount)	12-13
Outcome data	15*	Report numbers of outcome events or summary measures over time	13

© 2024 Friesen EL et al. *JAMA Network Open.*

Main results	16	(*a*) Give unadjusted estimates and, if applicable, confounder-adjusted estimates and their precision (eg, 95% confidence interval). Make clear which confounders were adjusted for and why they were included<br>(*b*) Report category boundaries when continuous variables were categorized<br>(*c*) If relevant, consider translating estimates of relative risk into absolute risk for a meaningful time period	14
Other analyses	17	Report other analyses done—eg analyses of subgroups and interactions, and sensitivity analyses	NA
**Discussion**			
Key results	18	Summarise key results with reference to study objectives	14-15
Limitations	19	Discuss limitations of the study, taking into account sources of potential bias or imprecision. Discuss both direction and magnitude of any potential bias	16-17
Interpretation	20	Give a cautious overall interpretation of results considering objectives, limitations, multiplicity of analyses, results from similar studies, and other relevant evidence	14-18
Generalisability	21	Discuss the generalisability (external validity) of the study results	14-18
**Other information**			
Funding	22	Give the source of funding and the role of the funders for the present study and, if applicable, for the original study on which the present article is based	18

*Give information separately for exposed and unexposed groups.

Note: An Explanation and Elaboration article discusses each checklist item and gives methodological background and published examples of transparent reporting. The STROBE checklist is best used in conjunction with this article (freely available on the Web sites of PLoS Medicine at http://www.plosmedicine.org/, Annals of Internal Medicine at http://www.annals.org/, and Epidemiology at http://www.epidem.com/). Information on the STROBE Initiative is available at http://www.strobe-statement.org.

© 2024 Friesen EL et al. *JAMA Network Open.*
